# History of the Epidemic Yellow Fever

**Published:** 1854-04

**Authors:** E. D. Fenner


### *History of the Epidemic Yellow Fever at New Orleans,* 1853.

BY E. D. FENNER, M. D.

This is a pamphlet of 64 pages, and we acknowlede the favor of a copy for which we are under obligations to the author. The work is highly interesting and is a valuable contribution to science upon the subject of which it treats. The opportunities of the author for observation and experiment have enabled him to collect and collate many valuable facts touching its nature as well as the mode of treating it. These he has presented in the work for the benefit of the profession, particularly those of its members residing in those localities liable to its visitations.

We would be pleased to extract liberally from this little offering, but will content ourselves, for the present, with the following "Death and

Dr. Hornbrook" communication, which first appeared in the Delta, a secular journal of N. O. Dr. F. transferred it to the pages of his work, and we copy it because, in its own peculiar way, it points its quill at the culpable remissness of the "fathers" of the devoted "Crescent City," in relation to certain sanatory measures, and from which the hoary-headed paternity of other cities may find some valuable hints. Some of our northern cities may find abundant reason of self-gratulation that the "old gentleman" had so much "business on his hands" in the South, for had he been allowed the privilege of a visit "north," he would have found our cities reposing in self-security, with abundant material on hand to make keen the edge of his scythe. The exemption from cholera brought about an apathy upon the subject of sanatory regulations, which neglect, judging from reports, has been carried to a criminal extent. Dr. Rush maintained that the city authorities were chargeable with the deaths of those which took place and which could be traced to a cause susceptible of removal or abatement, and it was his deliberate opinion that they were justly liable to a prosecution at law. We have ever held that measures looking to the health of the citizens, are second in importance to none other in the improvements of a city, on the contrary, they should be the first entered upon and persisted in.

ANTI-YELLOW FEVER.

(From the Delta, June 22, 1853.) (Communicated.)

EDS. DELTA—I wish to relate to you a very strange affair which happened to me a few evenings since:

I had driven to the Lake with a friend, and becoming wearied of the place, and he being engaged and likely to stay some time longer, I concluded to walk at my leisure towards town, until he caught up with me with the buggy.

It was twilight, and as I walked slowly, musing over the past, and imagining the future of the great city before me, I saw just ahead, seated upon a stump by the side of the road, one of the queerest little figures you can conceive of. His back was towards me, and he was bent down, very busily engaged at something. As I came to his side I saw that he was sharpening a scythe.

The appearance and occupation of the little old gentleman were so strange, that I stopped to examine him more thoroughly. He had on an old yellow coat, quite voluminous in the body, but with very short sleeves. His trowsers were of the thunder-and-lightning pattern, well shrunk up, and short enough to please the veriest dandy in town. His shirt had a large collar, but was without plaits in the bosom. In fine, his clothes were of antiquated material and cut, quite clean, but neither ironed nor starched.

But what attracted my attention most was his face. His features were very small, pinched and wrinkled, something like old and rumpled parchment. His sharp nose, the quick movements of his bony hands, and the twisting of his puckered mouth as he worked with his hone, denoted nervousness.

At last he got through his job, and passing his finger over the edge to try its keenness, gave a grunt of satisfaction, and looking up, his keen ferret-eyes looking full into mine, he ejaculated—"Ah ha, is that you?" with such suddenness and sharpness, that I was quite startled.

"Dull times. But that's nice, isn't it?" said he, snuffing with great satisfaction the foul wind coming from the direction of the city.

I differed with him, and received for my pains a sharp humph! accompanied with a look of contempt.

"Why," said he, "it's the very thing, and it will be better than that in a few days."

Thinking the old gentleman a little cracked, I ventured to ask him what he was going to do with his scythe.

"Going to work," said he, getting off the stump and stretching himself.

"But there's no grass for you to mow down here."

"No," said he, "I am going into the city to work."

"But there is no grass in the city either," I suggested.

"Oh, plenty, plenty," said he, with a chuckle. "Plenty of my sort of grass; men, sir, men. All flesh is grass, you know. Eh?"

"The Devil!" I blustered out in my astonishment, for I seldom use so strong language.

"No, sir, not the Devil, either; only a friend of his—an old friend. In fact, sir, some people say he is my father. Don't speak disrespectfully of my respected and venerable ancestor, I beg of you," said he, looking askance at me.

"You are Death, then," I tremblingly said, half interrogatively, half affirmatively.

"Yes, sir," said he, evidently enjoying my fright. "Yes, sir, at your service."

"Very much obliged," said I, thinking it best to appear brave; "but I have no present need for your services."

"No, of course not," said he, with a little severity in his tone.—"That is the usual answer. I did not suppose you had any use for me;" and he muttered something about jackanapes, and he wouldn't have asked me, and so on.

"Well," said the old gentleman, after musing awhile with his chin resting on the handle of his scythe, "well, isn't it a shame?"

I did not understand what he was alluding to. It was evident his mind had got into a new channel; so I asked, "Isn't what a shame?"

"Ain't you astonished at my personal appearance?" said he, looking himself over with evident pleasure; "you thought I was a naked skeleton. Come, now, didn't you? Speak the truth and shame the ——, and soforth, ahem? Yes, they pictured me as a skeleton, the impertinent rascals, a long time ago and I have never been able to stop the burlesque. I killed the fellows, sir, but the Press is a great disseminator of falsehood." And the old gentleman rested his chin on his scythe handle again, and muttering, "Never mind, what I have lost my respectable father has gained, and it's all in the family"—lapsed into the musing.

"Why do you come to New Orleans this summer?" said I breaking the silence; "it has been so long since you were here that they don't expect you."

"Young man," said Death, solemnly looking up, "don't talk so like a gump. I do just as you do—follow my nose; and if they don't expect me, more fools they. I like variety, and have an eye to future trade, so I didn't intend coming to New Orleans this summer. I was going North with the other fashionables; but your City Council have broken into my arrangements, and the people will have to suffer."

"But," said I, remonstrating, "how have the City Council broken into your arrangements? And you are not going to make the people suffer for what they have done? Surely they alone should bear it."

"I told you just now," said he, in a very positive manner, "that I follow my nose. If men will live in filth, will make a pest-house for themselves, *que voulez vous, mon cher?* I must act naturally. As for making the people suffer for the acts of their representatives, I do not see any wrong in that. All of us act upon that principle. But as for killing the City Council, there is no use of my troubling myself"

And here Death put up his hand to his mouth, and throwing his head back, gave me a leer, and imitated drinking out of a bottle.—Then he broke out into the most outrageous screeching laughter.

"Why, sir," said he, as soon as he had recovered from a fit of coughing, brought on by his cachinations, "I have heard some envious fellows say that the reason why the Council did not make their subs let the water flow through the gutters was, that they are interested in lots in the swamp, and are afraid they might be flooded. I have heard others say that they were so anxiously electioneering for the next Council, that they had neither time nor disposition to look after the Street Commissioners. Others say that since the city has been consolidated, they are not competent to attend to their other huge labors, let alone the care of gutters and sinks."

"Well, what do you think about it?" said I.

"What do I think? I think it is a little of all these reasons, and a few more besides—one of which is, that they are a careless set of fellows."

I do not wish to detail to you, Mr. Editor, all the conversation we had, though he told me some very curious things. He told me, at parting, that he wished the Council would do their duty, and let him go North, as he had no disposition to stay here. I was a little offended several times at the disrespectful manner in which he spoke of our council; but, as he said, *que voulez vous, mon cher?* It was the truth, and I had to admit it.

ANTI-YELLOW FEVER.

